# Age of Complementary Foods Introduction and Risk of Anemia in Children Aged 4–6 years: A Prospective Birth Cohort in China

**DOI:** 10.1038/srep44726

**Published:** 2017-03-23

**Authors:** Fenglei Wang, Huijuan Liu, Yi Wan, Jing Li, Yu Chen, Jusheng Zheng, Tao Huang, Duo Li

**Affiliations:** 1 Department of Food Science and Nutrition, Zhejiang University, Hangzhou, China; 2 Jiaxing Maternity and Child Health Care Hospital, Jiaxing, China; 3 MRC Epidemiology Unit, University of Cambridge, Cambridge, UK; 4 Saw Swee Hock School of Public Health, National University of Singapore, Singapore; 5 Institute of Nutrition and Health, Qingdao University, Qingdao, China

## Abstract

Age of complementary foods introduction is associated with childhood anemia, but the ideal age for the introduction of complementary foods to infants is a continuing topic of debate. We examined the longitudinal association between complementary foods introduction age and risk of anemia in 18,446 children from the Jiaxing Birth Cohort, who had detailed complementary feeding records at 3 and 6 months of age and had hemoglobin concentrations measured at 4–6 years. Early introduction of complementary foods at 3–6 months of age was significantly associated with a higher risk of anemia (odds ratio = 1.14; 95% confidence interval: 1.01–1.28) and a lower hemoglobin concentration of −0.84 g/L (95% confidence interval: −1.33 to −0.35) in children aged 4–6 years, compared with those fed complementary foods starting at 6 months of age. When it comes to the specific type of complementary foods, early introduction of all plant-based foods was associated with increased anemia risks and lower hemoglobin concentrations, while early introduction of most animal-based foods was not. These findings may be informative regarding the appropriate time to introduce complementary foods in infants.

Anemia, an indicator of both poor nutrition and poor health, is associated with reduced cognition and impaired physical capacity and work performance^1^,^2^. A recent report estimated that global anemia prevalence in 2013 was 27%, with the highest rate among children less than 5 years of age being roughly 43%, translating to 273 million children with anemia^3^. Previous documents from the World Health Organization (WHO) suggested nearly half of the world’s anemia burden arises from iron deficiency^4^. A meta-analysis of 33 randomized controlled trials found that daily oral iron could effectively reduce the risk of anemia in children aged 4–23 months^5^. Addressing iron deficiency has been considered one of the most effective interventions for preventing and controlling anemia^6^,^7^.

There is universal agreement that human milk alone is the optimal first food for infants^8^. Although the concentration of iron in human milk is relatively low, the bioavailability is high, frequently cited as 12–56%^9^,^10^. Nevertheless, with the growth of infants and concomitant expansion of blood volume, iron intake from human milk is unlikely to be sufficient to meet the increasing needs of body tissue and circulation^9^,^11^. Therefore iron-rich complementary foods should be given in addition to human milk to fill the gap at the proper time^12^,^13^. However, the ideal age for the introduction of complementary foods to infants is a continuing topic of debate. For the past few decades in the last century, WHO recommended that complementary foods should be started at 4 to 6 months of age^14^. In contrast, at the beginning of this century, after an expert consultation on complementary feeding, WHO changed the recommended age for introduction of complementary foods to 6 months of age^15^. Studies on the association between age of complementary foods introduction and iron status or anemia risk of young children have also produced mixed results. One randomized trial investigated the effect of timing for the introduction of complementary foods on iron status of infants^16^. It was observed that infants introduced to complementary foods at 3 to 4 months of age had greater mean iron intake than those who were fed complementary foods at 6 months of age; but no difference was found in their iron status. Meanwhile, a recently published meta-analysis showed higher hemoglobin and ferritin levels in infants fed complementary foods at 4 months compared with those fed at 6 months, suggesting iron deficiency anemia risk in infants could be positively altered by earlier introduction of complementary foods^17^.

The inconsistent results from few studies carried out on the association between timing of complementary foods introduction and anemia risk in young children warrant the need for more prospective studies. The primary aim of the present study was to examine the relationship between the age of complementary foods introduction and risk of anemia in children aged 4–6 years in Mainland China.

## Results

### Child and Maternal Characteristics According to Age of Complementary Foods Introduction

Among the 18,446 children, 13.1% were introduced to complementary foods at 3 to 6 months of age and 86.9% after 6 months of age. Children born to mothers having a higher education or whose occupation was not farming were less likely to be fed complementary foods before 6 months. No significant difference was found in children’s sex, birth weight, breastfeeding status, maternal body mass index and hemoglobin concentrations at first screening between two groups; while children’s age at follow-up and other characteristics of the mothers (age at delivery, folic acid supplementation, parity and cesarean delivery) varied between two groups (Table 1).

### Age of Complementary Foods Introduction and Risk of Anemia

Overall anemia incidence in children aged 4–6 years was 14.3%. In crude analysis, earlier introduction of complementary foods was significantly associated with anemia (OR: 1.27; 95% CI: 1.13–1.42), compared with introducing complementary foods from 6 months of age. The association of age of complementary foods introduction with anemia remained significant after adjustment for covariates of children’s characteristics (OR: 1.24; 95% CI: 1.11–1.40) or both children’s and maternal characteristics (OR: 1.14; 95% CI: 1.01–1.28) (Table 2). In the analysis stratified by children’s birth weight and maternal hemoglobin in early pregnancy, no significant interactions between age of introduction of complementary foods and these two factors were observed (all *P*-values for interaction tests >0.05) (Supplementary Table S1). Among the nine kinds of complementary foods, early introductions of bread/steamed bun/fine dried noodle, pureed noodle/cookies, tofu, and ground meat/soy product were significantly associated with anemia, whereas other foods were not found to be associated with anemia (Table 3).

### Age of Complementary Foods Introduction and Hemoglobin Concentration

The mean hemoglobin concentration for children aged 4–6 years was 125.3 ± 11.3 g/L. Hemoglobin was −1.19 g/L lower (95% CI: −1.68 to −0.70) among children receiving complementary foods at 3 to 6 months; the difference remained significant after adjusting for child and maternal covariates (mean difference = −0.84 g/L; 95% CI: −1.33 to −0.35) (Table 4). No significant interaction between complementary foods introduction age and birth weight and maternal hemoglobin in early pregnancy was found in stratified analyses (all *P*-values for interaction tests >0.05) (Supplementary Table S2). Similarly, among the complementary foods commonly used, significant associations between age of the introduction of complementary foods and hemoglobin concentrations were observed for rice cereal/porridge, bread/steamed bun/fine dried noodle, pureed noodle/cookies, tofu, egg yolk, and ground meat/soy product (Table 5). Children fed these foods at 3 to 6 months had significantly lower hemoglobin concentrations compared with those who were fed these foods after 6 months of age.

## Discussion

In this large prospective birth cohort study, we found that early introduction of complementary foods before 6 months was significantly associated with a greater risk of anemia and a lower hemoglobin concentration in children aged 4–6 years, even after adjustment for other risk factors like birth weight and maternal hemoglobin concentration. It is consistent with the current WHO recommendation of introducing complementary foods at 6 months. We also found that early introduction of plant-based foods such as rice cereal, porridge, bread, pureed noodle, and tofu, was more likely to be associated with increased risk of anemia and lower hemoglobin concentrations than animal-based foods.

Few studies have directly compared anemia risk of infants given complementary foods starting at different ages, and the results have been inconsistent. In an Italian study with a small number of infants, the prevalence of anemia at 12 months was significantly higher among those who were given complementary foods before 7 months than those who were exclusively breast-fed for ≥7 months, implying that early introduction of complementary foods was associated with anemia^18^. In contrast, a cross-sectional study conducted in rural areas of China, which investigated anemia prevalence in children introduced to complementary foods before 6 months and after 6 months showed no differences after adjusting for potential confounders^19^.

A number of previous studies have examined the relationship between age of complementary foods introduction and hemoglobin concentrations. A randomized controlled trial conducted in Honduras investigated the effects of age for introduction of complementary foods on iron status of infants^20^. Children who received complementary foods from 4 months of age had a higher hemoglobin value at 6 months than those exclusively breastfed for 6 months. In contrast, no significant difference in hemoglobin was detected in another similarly designed trial conducted in a high-income country, Iceland^21^. One trial performed in America, also found no effect in the timing of introduction of complementary foods on hemoglobin concentrations of infants at 12, 24, and 36 months of age^16^. By contrast, our study observed that children receiving complementary foods at 3–6 months had lower hemoglobin concentrations at 4–6 years of age. The complementary foods used in previous and the present studies varied. Complementary foods in the Honduran study included iron-fortified rice cereal and iron-fortified rice cereal with egg yolk, while foods in the present study were all non-iron-fortified, which may have contributed to the different results in hemoglobin concentration.

The age of when complementary foods are introduced may become critically important when infants are not fed an external source of iron. In a Chilean study, the effect of iron-fortified rice cereal in preventing iron deficiency was compared with unfortified rice cereal in infants who were exclusively breast-fed for more than 4 months^22^. There was a greater prevalence of iron deficiency anemia in infants who were fed unfortified rice cereal than those receiving iron-fortified rice cereal. Similarly, a randomized controlled trial conducted in South Africa, assessed whether the fortified maize-meal porridge could reduce anemia of infants^23^. It found that the proportion of infants with anemia was significantly decreased in the fortified-porridge group while it remained unchanged in the unfortified-porridge group.

Amongst the different types of complementary foods investigated in the present study, early introduction of plant-based foods, namely rice cereal, porridge, bread, pureed noodle, and tofu, was associated with an increased risk of anemia and a lower hemoglobin concentration, while introduction of most animal-based food was not. In addition, of those who were fed complementary foods before 6 months of age, 27% children received plant-only-based foods, mainly grains and cereals. These plant-based foods often contain low amounts of bioavailable iron, and will also contain components, which will inhibit non-heme iron absorption, such as phytates^24^. It has been generally accepted that plant-based complementary foods alone are inadequate to meet the needs of iron and other nutrients such as zinc and calcium, without the use of supplements or nutrient-fortified foods^15^,^25^.

The main strength of this study is that it is the first and largest longitudinal study reported so far in China or elsewhere in the world that investigated the association between age of complementary foods introduction and anemia in children aged 4–6 years. Longitudinal investigation of incident anemia could provide stronger causal evidence than cross-sectional analyses. Furthermore, the association we obtained was robust in multiple sensitivity analyses. We observed similar results after adjustment for many important child and maternal characteristics related to anemia, for instance, birth weight, deliver type, and maternal hemoglobin concentration. We also performed a sensitivity analysis that omitted children with incomplete covariate data and the results were almost unchanged (Supplementary Tables S3 and S4). In addition, we also carefully collected information on the time when specific types of complementary foods were introduced, which made it possible to evaluate whether the associations between different kinds of complementary foods and anemia varied.

Nonetheless, a few potential limitations need to be addressed. Firstly, timing of umbilical cord clamping could have some effect on blood volume and hence total body iron content of infants^9^. Unfortunately, we did not collect data about cord clamping procedures. Secondly, we also lacked detailed information regarding the frequency of human milk or formula feeding and other supplementation records during infant period, amount of complementary foods, children’s partial eclipse behavior and infectious diseases, which may have influenced the study outcomes. Thirdly, all the complementary foods we studied were non-fortified, meaning that the findings of this study are not generalizable to those fed iron-fortified foods. However, in underdeveloped areas like rural central and western China^26^ and other developing countries, infants are generally poorly fed, with mostly plant-based foods and rarely iron-fortified foods. Therefore, our results are of practical importance to these areas. Finally, we did have losses to follow-up. Because of the large sample size and participants scattering around the Jiaxing area, it was hard to keep in touch with all these participants. Nevertheless, children included in the final analysis and those lost to follow up were similar with respect to sex, birth weight, breastfeeding status, and maternal education, occupation, folic acid supplementation, BMI, hemoglobin concentration at first screening and caesarean delivery (Supplementary Table S5).

## Conclusions

In summary, early introduction of complementary foods, especially iron-unfortified plant-based foods before 6 months was significantly associated with a greater risk of anemia and a lower hemoglobin concentration in children aged 4–6 years. These findings are consistent with WHO recommendations on the age of complementary foods introduction for infants and suggest that appropriate complementary feeding should start from the age of 6 months. Furthermore, large well-designed prospective research studies are needed, particularly in countries where infants are not fed appropriate complementary foods to determine the consequences associated with childhood anemia.

## Methods

Ethics approval of this study was obtained from the Ethics Committee of the College of Biosystem Engineering & Food Science at Zhejiang University (Approval Number 2013013). All procedures performed were in accordance with the approved guidelines. All of the parents gave their oral informed consent.

### Study Subjects

Initiated in 1993, Jiaxing Birth Cohort was part of a large population-based health surveillance system in China and has been described previously^27^,^28^. Briefly, pregnant women in Jiaxing city of Zhejiang Province in Southeast China were recruited when visiting local clinics or maternity and child health care hospitals. General information was collected at their first visit and they were asked to visit the clinics or hospitals after birth, until their children were 5–6 years of age. Subsequent follow-up information regarding their children’s feeding practice, hemoglobin, behavioral and anthropometric parameters were obtained at each visit.

During the period of 1999 to 2009, 50,574 singleton children included in the Jiaxing Birth Cohort provided complementary feeding information and hemoglobin record at 3 months of age. Of these, 1558 were excluded because of extreme values of gestational week (<37 or >44 weeks), and 615 were excluded due to extreme birth weight (<2500 or ≥5000 g). We also excluded children born to mothers without hemoglobin records in early pregnancy (before 20 weeks of gestation) (n = 2236) or whose mothers had potentially spurious hemoglobin values (<60 or >200 g/L; n = 149). Additionally, children introduced to complementary foods before or at 3 months of age (n = 551) were further excluded because at this period, infants haven’t reached sufficient maturity of renal and gastrointestinal function to tolerate complementary foods^29^,^30^. To calculate the incidence of anemia during the follow-up period, children who were anemic at 3 months of age were also excluded (n = 14,771). After these exclusions, 30,694 children were included as baseline. Detailed feeding information of these children was then recorded at 6 months of age and 18,457 of them had their hemoglobin measured at 4–6 years of age. After excluding children having a potentially spurious hemoglobin value (n = 11), a total of 18,446 children were included in the final analysis.

### Complementary Feeding Information and Key Study Variables

At each visit when children were 3 and 6 months of age, parents were asked by a trained nurse whether their children had been introduced to the following nine kinds of food commonly consumed in Southeast China: rice cereal/porridge, bread/steamed bun/fine dried noodle, pureed noodle/cookies, tofu, egg yolk, fish paste, liver paste, animal blood, and ground meat/soy product. Children were divided into 2 groups based on the age of complementary foods introduction: “3–6 months” (if they were fed complementary foods between 3 to 6 months of age) and “≥6 months” (if they received complementary foods starting from 6 months of age).

According to previous publications^31^, other covariates were also collected during the follow-up visits. Continuous covariates included child’s birth weight and age at follow-up, and maternal age at delivery, body mass index (BMI), and hemoglobin in early pregnancy (before 20 weeks of gestation). Categorical covariates included child’s sex, breastfeeding status (never breastfed, primarily breastfed, or exclusively breastfed), maternal education (<high school, high school, and >high school), occupation (farmer or other), folic acid supplementation (yes or no), parity (primigravida or multigravida) and caesarean section (yes or no).

### Outcome Measures and Anemia Definition

Each year when children were 4–6 years of age, hemoglobin levels were measured in a blinded fashion at their visit to the local clinics or hospitals, with a standard cyanmethemoglobin method by using capillary blood. For those who had their hemoglobin measured more than once, the first measurement value was used to avoid confounding effects produced after the first measurement. Following WHO recommendations, anemia was defined as hemoglobin concentrations <110 g/L for children aged <60 months and <115 g/L for children aged ≥60 months^32^.

### Statistical Analyses

Child and maternal characteristics by age of complementary foods introduction were assessed by chi-square test. Odds ratio (OR) was used to assess the association between age of complementary foods introduction and risk of anemia. Crude ORs of anemia for children fed complementary foods at different ages were estimated by univariate logistic regression. Adjusted ORs were then estimated using multiple logistic regression. Association between age of complementary foods introduction and hemoglobin concentration of young children were assessed via linear regression. Covariates included in these statistical models were as follows: child’s age (<60 months or ≥60 months), sex (male or female), birth weight (2500–2999, 3000–3499, 3500–3999, and ≥4000 g), breastfeeding status (never, primarily or exclusively), maternal age at delivery (<25, 25–29, and ≥30 years), education (<high school, high school, and >high school), occupation (farmer or other), folic acid supplementation (yes or no), BMI (<18.5, 18.5–24.9, and ≥25 kg/m^2^), hemoglobin in early pregnancy (<110, 110–119, 120–129 and ≥130 g/L), parity (primigravida or multigravida) and caesarean section (yes or no). Percentages of missing data on maternal BMI, education, occupation, folic acid supplementation and caesarean section were 0.7%, 0.1%, 0.1%, 0.6% and 0.3%, respectively. To examine the impact of missing data on our analysis, a sensitivity analysis was conducted by omitting those children with these missing data. Stratified analyses based on child’s birth weight and maternal hemoglobin status were performed to examine their influence on the association of timing of complementary foods introduction with risk of anemia, according to previous studies^9^,^31^. The association between particular type of complementary foods and anemia risk was also examined using multiple logistic regression. All statistical analyses were carried out using SAS (version 9.3) for Windows. Except where otherwise specified, a two-tailed *P*-value < 0.05 was considered significant.

## Additional Information

**How to cite this article:** Wang, F. *et al*. Age of Complementary Foods Introduction and Risk of Anemia in Children Aged 4–6 years: A Prospective Birth Cohort in China. *Sci. Rep.* **7**, 44726; doi: 10.1038/srep44726 (2017).

**Publisher's note:** Springer Nature remains neutral with regard to jurisdictional claims in published maps and institutional affiliations.

## Supplementary Material

Supplementary Tables

## Figures and Tables

**Table 1:** Child and maternal characteristics according to age of complementary foods introduction.

	n	Age of Complementary Foods Introduction		*P*
		3–6 months	≥6 months	
Age at follow-up (month)				0.002
<60	3890	450 (18.6)	3440 (21.5)	
≥60	14,556	1964 (81.4)	12,592 (78.5)	
Sex				0.169
Male	9401	1262 (52.3)	8139 (50.8)	
Female	9045	1152 (47.7)	7893 (49.2)	
Birth weight (g)				0.513
2500–2999	2789	373 (15.5)	2416 (15.1)	
3000–3499	8868	1128 (46.7)	7740 (48.3)	
3500–3999	5507	735 (30.4)	4772 (29.8)	
≥4000	1282	178 (7.4)	1104 (6.9)	
Breastfeeding status				0.855
Never	1367	181 (7.5)	1186 (7.4)	
Primarily	5184	667 (27.6)	4517 (28.2)	
Exclusively	11,895	1566 (64.9)	10,329 (64.4)	
Mother’s age at delivery (year)				<0.001
<25	10,949	1537 (63.7)	9412 (58.7)	
25–29	5229	590 (24.4)	4639 (28.9)	
≥30	2268	287 (11.9)	1981 (12.4)	
Maternal education				<0.001
<High school	13,152	2050 (85.0)	11,102 (69.3)	
High school	3480	283 (11.7)	3197 (20.0)	
>High school	1798	80 (3.3)	1718 (10.7)	
Maternal occupation				<0.001
Farmer	11,993	1840 (76.3)	10,153 (63.4)	
Other	6442	573 (23.7)	5869 (36.6)	
Folic acid supplementation				0.001
Yes	2122	230 (9.6)	1892 (11.9)	
No	16,218	2168 (90.4)	14,050 (88.1)	
BMI at first screening (kg/m^2^)				0.466
<18.5	3298	1420 (41.7)	2878 (18.1)	
18.5–24.9	13,757	1807 (53.1)	11,950 (75.1)	
≥25	1261	178 (5.2)	1083 (6.8)	
Hemoglobin at first screening (g/L)				0.140
<110	3061	426 (17.6)	2635 (16.4)	
110–119	5672	747 (30.9)	4925 (30.7)	
120–129	5560	683 (28.3)	4877 (30.4)	
≥130	4153	558 (23.1)	3595 (22.5)	
Parity				<0.001
Primigravida	15,654	1953 (80.9)	13,701 (85.5)	
Multigravida	2792	461 (19.1)	2331 (14.5)	
Caesarean delivery				0.010
Yes	13,644	1730 (72.1)	11,914 (74.5)	
No	4747	671 (27.9)	4076 (25.5)	

Data are presented as number (column percentage) of children unless otherwise indicated.

**Table 2:** Crude and adjusted odds ratio of anemia for introducing complementary foods at 3–6 months compared with ≥6 months.

	Risk of Anemia	
	3–6 months	≥6 months
Case/Study participants	409/2414	2222/16,032
Crude OR (95% CI)	1.27 (1.13–1.42)	1
Adjusted OR (95% CI)^a^	1.24 (1.11–1.40)	1
Adjusted OR (95% CI)^b^	1.14 (1.01–1.28)	1

OR, odds ratio; CI: confidence interval.

^a^OR was adjusted for child’s age, sex, birth weight and breastfeeding status.

^b^OR was adjusted for child’s age, sex, birth weight and breastfeeding status, and maternal characteristics (age at delivery, education, occupation, folic acid supplementation, BMI, hemoglobin in early pregnancy, parity and caesarean section).

**Table 3:** Crude and adjusted odds ratio of anemia for introducing different types of complementary foods at 3–6 months compared with ≥6 months.

	Risk of Anemia	
	3–6 months	≥6 months
Rice cereal/porridge		
Case/Study participants	367/2187	2264/16,259
Crude OR (95% CI)	1.24 (1.10–1.40)	1
Adjusted OR (95% CI)	1.12 (0.99–1.26)	1
Bread/steamed bun/fine dried noodle		
Case/Study participants	122/604	2509/17,842
Crude OR (95% CI)	1.55 (1.27–1.90)	1
Adjusted OR (95% CI)	1.38 (1.12–1.69)	1
Pureed noodle/cookies		
Case/Study participants	122/633	2509/17,813
Crude OR (95% CI)	1.46 (1.20–1.79)	1
Adjusted OR (95% CI)	1.29 (1.05–1.58)	1
Tofu		
Case/Study participants	215/1169	2416/17,277
Crude OR (95% CI)	1.38 (1.18–1.61)	1
Adjusted OR (95% CI)	1.21 (1.03–1.41)	1
Egg yolk		
Case/Study participants	275/1641	2356/16,805
Crude OR (95% CI)	1.23 (1.07–1.41)	1
Adjusted OR (95% CI)	1.11 (0.96–1.27)	1
Fish paste		
Case/Study participants	198//1155	2433/17,291
Crude OR (95% CI)	1.26 (1.07–1.48)	1
Adjusted OR (95% CI)	1.12 (0.95–1.32)	1
Liver paste		
Case/Study participants	101/596	2530/17,850
Crude OR (95% CI)	1.24 (1.00–1.54)	1
Adjusted OR (95% CI)	1.09 (0.87–1.36)	1
Animal blood		
Case/Study participants	114/700	2517/17,746
Crude OR (95% CI)	1.17 (0.95–1.44)	1
Adjusted OR (95% CI)	1.04 (0.84–1.28)	1
Ground meat/soy product		
Case/Study participants	123/604	2508/17,842
Crude OR (95% CI)	1.57 (1.28–1.92)	1
Adjusted OR (95% CI)	1.39 (1.14–1.71)	1

OR, odds ratio; CI: confidence interval.

OR was adjusted for child’s age, sex, birth weight and breastfeeding status, and maternal characteristics (age at delivery, education, occupation, folic acid supplementation, BMI, hemoglobin in early pregnancy, parity and caesarean section).

**Table 4:** Crude and adjusted mean differences in hemoglobin (g/L) for introducing complementary foods at 3–6 months compared with ≥6 months.

	Hemoglobin Concentration	
	3–6 months	≥6 months
Mean ± SD	124.3 ± 12.1	125.5 ± 11.2
Unadjusted MD (95% CI)	−1.19 (−1.68 to −0.70)	0
Adjusted MD (95% CI)^a^	−1.25 (−1.73 to −0.76)	0
Adjusted MD (95% CI)^b^	−0.84 (−1.33 to −0.35)	0

MD, mean difference; CI: confidence interval.

^a^MD was adjusted for child’s age, sex, birth weight and breastfeeding status.

^b^MD was adjusted for child’s age, sex, birth weight and breastfeeding status, and maternal characteristics (age at delivery, education, occupation, folic acid supplementation, BMI, hemoglobin in early pregnancy, parity and caesarean section).

**Table 5:** Crude and adjusted mean differences in hemoglobin (g/L) for introducing complementary foods at 3–6 months compared with ≥6 months.

	Hemoglobin Concentration	
	3–6 months	≥6 months
Rice cereal/porridge		
Mean ± SD	124.4 ± 12.2	125.5 ± 11.2
Unadjusted MD (95% CI)	−1.07 (−1.58 to −0.56)	0
Adjusted MD (95% CI)	−0.71 (−1.22 to −0.20)	0
Bread/steamed bun/fine dried noodle		
Mean ± SD	123.8 ± 13.1	125.4 ± 11.3
Unadjusted MD (95% CI)	−1.63 (−2.56 to −0.71)	0
Adjusted MD (95% CI)	−1.26 (−2.18 to −0.34)	0
Pureed noodle/cookies		
Mean ± SD	124.1 ± 12.7	125.4 ± 11.3
Unadjusted MD (95% CI)	−1.33 (−2.23 to −0.42)	0
Adjusted MD (95% CI)	−0.96 (−1.86 to −0.06)	0
Tofu		
Mean ± SD	123.9 ± 12.4	125.4 ± 11.3
Unadjusted MD (95% CI)	−1.53 (−2.21 to −0.86)	0
Adjusted MD (95% CI)	−1.17 (−1.85 to −0.50)	0
Egg yolk		
Mean ± SD	124.4 ± 12.2	125.4 ± 11.3
Unadjusted MD (95% CI)	−0.98 (−1.56 to −0.40)	0
Adjusted MD (95% CI)	−0.65 (−1.23 to −0.07)	0
Fish paste		
Mean ± SD	124.4 ± 12.3	125.4 ± 11.3
Unadjusted MD (95% CI)	−0.92 (−1.60 to −0.24)	0
Adjusted MD (95% CI)	−0.44 (−1.36–0.49)	0
Liver paste		
Mean ± SD	124.6 ± 12.6	125.4 ± 11.3
Unadjusted MD (95% CI)	−0.81 (−1.74–0.12)	0
Adjusted MD (95% CI)	−0.03 (−0.96–0.89)	0
Animal blood		
Mean ± SD	124.9 ± 12.4	125.4 ± 11.3
Unadjusted MD (95% CI)	−0.47 (−1.32–0.40)	0
Adjusted MD (95% CI)	−0.12 (−0.98–0.73)	0
Ground meat/soy product		
Mean ± SD	123.8 ± 12.6	125.4 ± 11.3
Unadjusted MD (95% CI)	−1.64 (−2.56 to −0.72)	0
Adjusted MD (95% CI)	−1.26 (−2.18 to −0.34)	0

MD, mean difference; CI: confidence interval.

MD was adjusted for child’s age, sex, birth weight and breastfeeding status, and maternal characteristics (age at delivery, education, occupation, folic acid supplementation, BMI, hemoglobin in early pregnancy, parity and caesarean section).
