# Supplementary material for: Age of Complementary Foods Introduction and Risk of Anemia in Children Aged 4–6 years: A Prospective Birth Cohort in China
Source: Sci Rep. 2017 Mar 23;7:44726. doi: 10.1038/srep44726 (PMC5363060; doi:10.1038/srep44726)
Supplement: Supplementary Tables [file srep44726-s1.pdf]

Age of Complementary Foods Introduction and Risk of Anemia in Children Aged 4-6 years: A Prospective Birth Cohort in China

Fenglei Wang, Huijuan Liu, Yi Wan, Jing Li, Yu Chen, Jusheng Zheng, Tao Huang, Duo Li*

Supplementary Table S1. Adjusted odds ratio of anemia for introducing complementary foods at 3-6 months compared with ≥6 months, stratified by child's birth weight and maternal hemoglobin at first screening.

	Risk of Anemia		*P* for interaction
	3-6 months	≥6 months	
Birth weight			0.802
2500-2999 g	1.10 (0.82-1.48)	1	
3000-3499 g	1.10 (0.93-1.31)	1	
3500-3999 g	1.25 (1.01-1.55)	1	
≥4000 g	1.08 (0.71-1.66)	1	
Hemoglobin at first screening			0.456
<110 g/L	1.10 (0.83-1.47)	1	
110-119 g/L	1.12 (0.91-1.40)	1	
120-129 g/L	1.05 (0.84-1.31)	1	
≥130 g/L	1.31 (1.03-1.65)	1	

Odds ratio was adjusted for child's sex, age, birth weight, breastfeeding status, and maternal characteristics (age at delivery, education, occupation, folic acid supplementation, BMI, hemoglobin at first screening before birth, parity and caesarean section).

Supplementary Table S2. Adjusted mean difference in hemoglobin (g/L) for introducing complementary foods at 3-6 months compared with ≥6 months, stratified by child's birth weight and maternal hemoglobin at first screening.

	Hemoglobin Concentration		*P* for interaction
	3-6 mon	≥6 mon	
Birth weight			0.452
2500-2999 g	-0.26 (-1.51-0.99)	0	
3000-3499 g	-0.73 (-1.45--0.02)	0	
3500-3999 g	-1.37 (-2.26--0.49)	0	
≥4000 g	-0.94 (-2.74-0.86)	0	
Hemoglobin at first screening			0.495
<110 g/L	-0.62 (-1.81-0.57)	0	
110-119 g/L	-0.68 (-1.56-0.20)	0	
120-129 g/L	-0.76 (-1.68-0.15)	0	
≥130 g/L	-1.34 (-2.35--0.34)	0	

Mean difference was adjusted for child's sex, age, birth weight, breastfeeding status, and maternal characteristics (age at delivery, education, occupation, folic acid supplementation, BMI, hemoglobin at first screening before birth, parity and caesarean section).

Supplementary Table S3. Crude and adjusted odds ratio of anemia for introducing complementary foods at 3-6 months compared with ≥6 months, after omitting children with incomplete covariate data.

	Risk of Anemia	
	3-6 months	≥6 months
Case/Study participants	405/2387	2195/15,797
Crude OR (95% CI)	1.24 (1.10-1.40)	1
Adjusted OR (95% CI)[a]	1.24 (1.10-1.40)	1
Adjusted OR (95% CI)[b]	1.14 (1.01-1.28)	1

OR, odds ratio; CI: confidence interval.

[a] OR was adjusted for child's age, sex, birth weight and breastfeeding status

[b] OR was adjusted for child's age, sex, birth weight and breastfeeding status, and maternal characteristics (age at delivery, education, occupation, folic acid supplementation, BMI, hemoglobin in early pregnancy, parity and caesarean section).

Supplementary Table S4. Crude and adjusted mean difference in hemoglobin (g/L) for introducing complementary foods at 3-6 months compared with ≥6 months, after omitting children with incomplete covariate data.

	Hemoglobin Concentration	
	3-6 months	≥6 months
Mean ± SD	124.3 ± 12.2	125.5 ± 11.2
Unadjusted MD (95% CI)	-1.19 (-1.68--0.70)	0
Adjusted MD (95% CI)[a]	-1.24 (-1.73--0.76)	0
Adjusted MD (95% CI)[b]	-0.84 (-1.33--0.35)	0

MD, mean difference; CI: confidence interval.

[a] MD was adjusted for child's age, sex, birth weight and breastfeeding status

[b] MD was adjusted for child's age, sex, birth weight and breastfeeding status, and maternal characteristics (age at delivery, education, occupation, folic acid supplementation, BMI, hemoglobin in early pregnancy, parity and caesarean section).

Supplementary Table S5. Child and maternal characteristic of those included in the final analyses (attendees) and those lost to follow up (non-attendees).

	n	Children at Baseline		*P*
		Attendees	Non-attendees	
Sex				0.521
Male	15,597	9401 (51.0)	6196 (50.6)	
Female	15,097	9045 (49.0)	6052 (49.4)	
Birth weight (g)				0.753
2500-2999	4595	2789 (15.1)	1806 (14.7)	
3000-3499	14,748	8868 (48.1)	5880 (48.0)	
3500-3999	9196	5507 (29.9)	3689 (30.1)	
≥4000	2155	1282 (7.0)	873 (7.1)	
Breastfeeding status				0.444
Never	2235	1367 (7.4)	868 (7.1)	
Primarily	8682	5184 (28.1)	3498 (28.6)	
Exclusively	19,777	11,895 (64.5)	7882 (64.4)	
Mother's age at delivery (year)				0.001
<25	18,425	10,949 (59.4)	7476 (61.0)	
25-29	8645	5229 (28.3)	3416 (27.9)	
≥30	3624	2268 (12.3)	1356 (11.1)	
Maternal education				0.178
<High school	21,781	13,152 (71.4)	8629 (70.5)	
High school	5837	3480 (18.9)	2357 (19.2)	
>High school	3058	1798 (9.8)	1260 (10.3)	
Maternal occupation				0.386
Farmer	19,900	11,993 (65.1)	7907 (64.6)	
Other	10,781	6442 (34.9)	4339 (35.4)	
Folic acid supplementation				0.070
Yes	3614	2122 (11.6)	1492 (12.3)	
No	26,897	16,218 (88.4)	10,679 (87.7)	
BMI at first screening (kg/m^2)				0.680
<18.5	5523	3337 (18.1)	2186 (17.8)	
18.5-24.9	22,944	13,757 (74.6)	9187 (75.0)	
≥25	2227	1352 (7.3)	875 (7.1)	
Hemoglobin at first screening (g/L)				0.168
<110	5170	3061 (16.6)	2109 (17.2)	
110-119	9302	5672 (30.7)	3630 (29.6)	
120-129	9293	5560 (30.1)	3733 (30.5)	
≥130	6929	4153 (22.5)	2776 (22.7)	
Parity				<0.001
Primigravida	25,835	15,654 (84.9)	10,181 (83.1)	
Multigravida	4859	2792 (15.1)	2067 (16.9)	
Caesarean delivery				0.287
Yes	22,635	13,644 (74.2)	8991 (73.6)	
No	7965	4747 (25.8)	3218 (26.4)	

Data are presented as number (column percentage) of children unless otherwise indicated.
